# Supplementary material for: Ocean tides trigger ice shelf rift growth and calving
Source: Nat Commun. 2025 Jul 24;16:6697. doi: 10.1038/s41467-025-61796-w (PMC12290053; doi:10.1038/s41467-025-61796-w)
Supplement: Supplementary file 1 — Supplementary Information [file 41467_2025_61796_MOESM1_ESM.docx]

**Supplementary Figures for “Ocean tides trigger ice shelf rift growth and calving”**


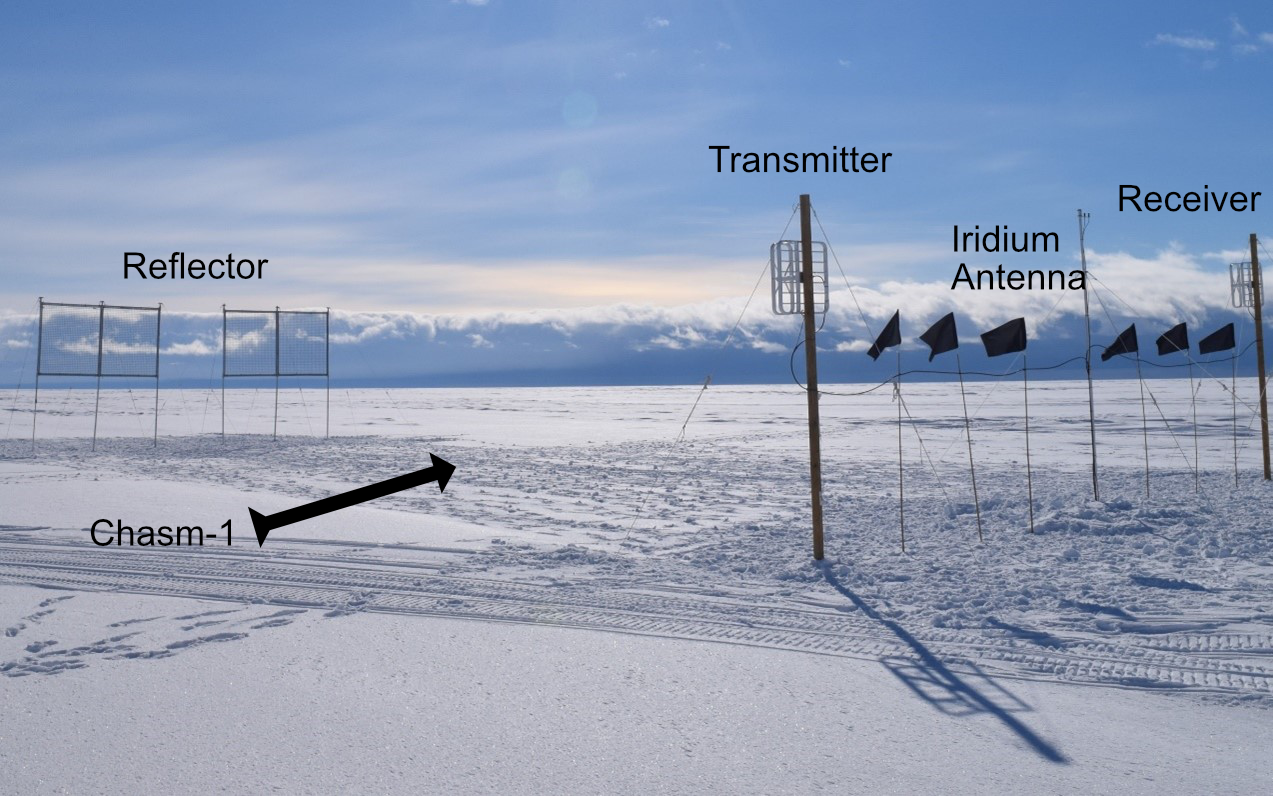


Figure S1: The ApRES set-up across Chasm-1 from January 2019.


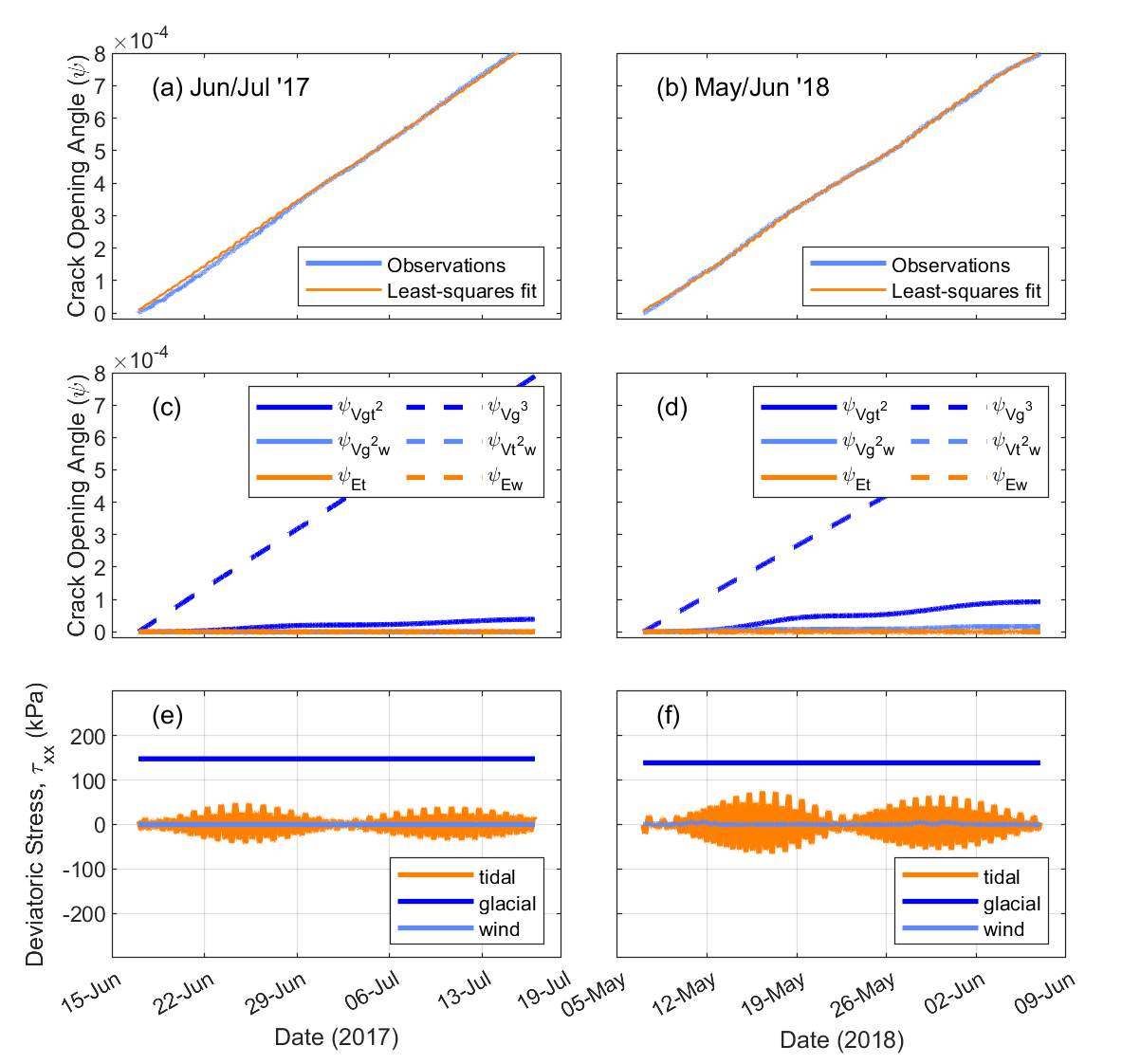


Figure S2: Same as Figure 2 but for ApRES data from 2017 and 2018.


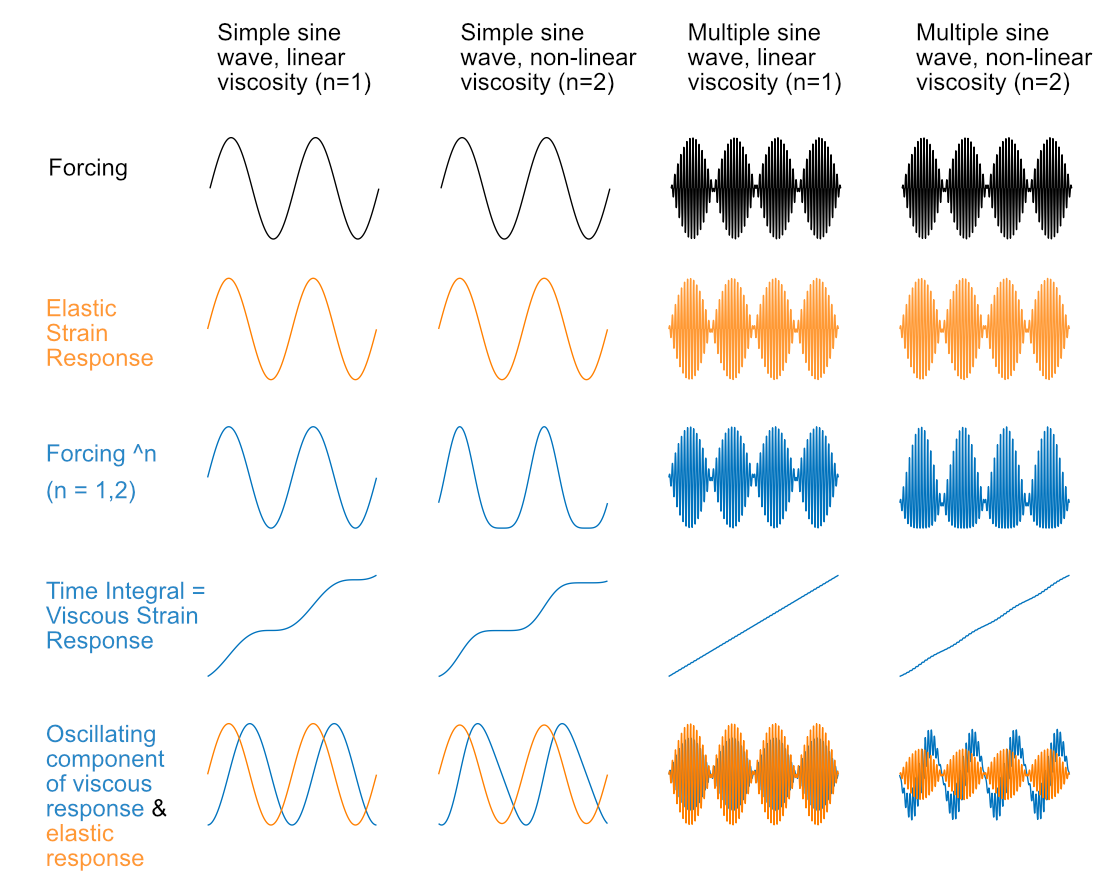


Figure S3: Schematic explanation of how the absolute magnitude of depth-averaged deviatoric stress can be determined from the relative strengths of the oscillating elastic and viscous responses due to their unique responses to the same forcing (bottom right). This only applies because there are oscillations at different frequencies from the tides and we have a non-linear viscosity.


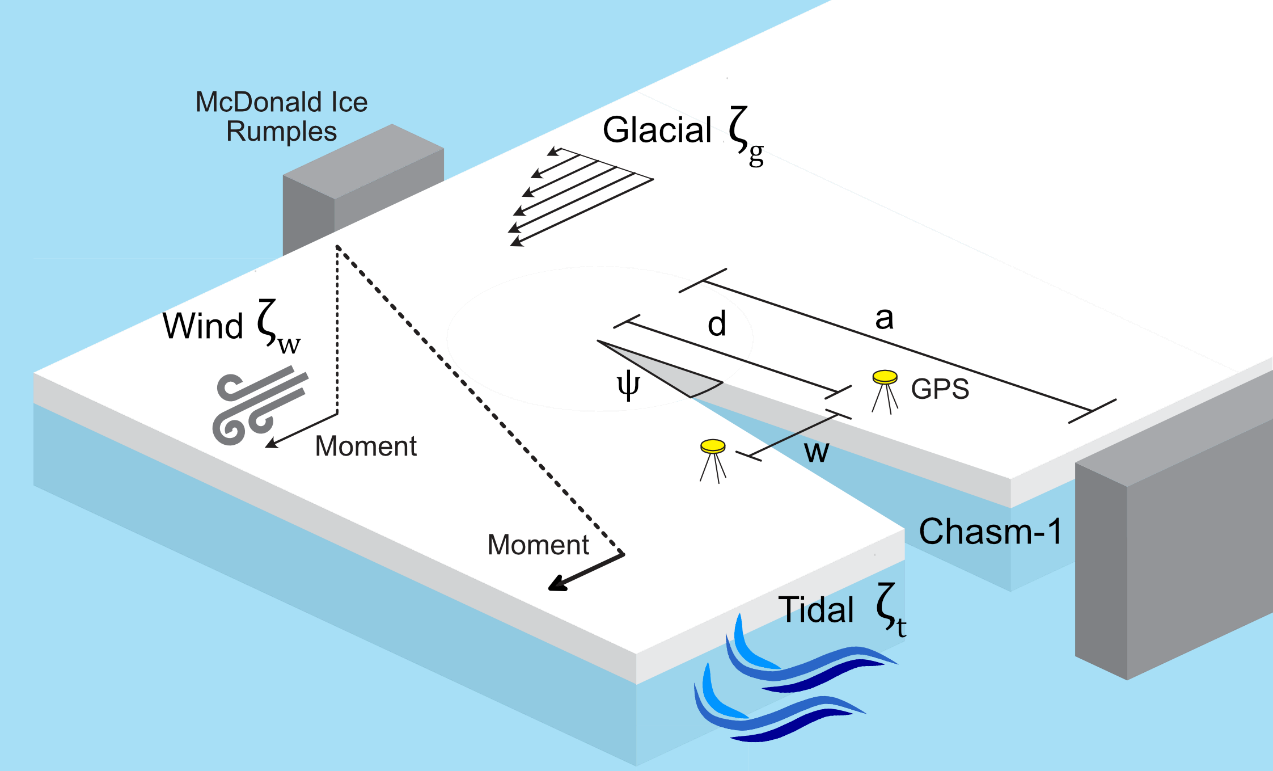


Figure S4: Schematic diagram showing enhancement of wind and tidal forcing as the rift grows due to the geometry of the rift.


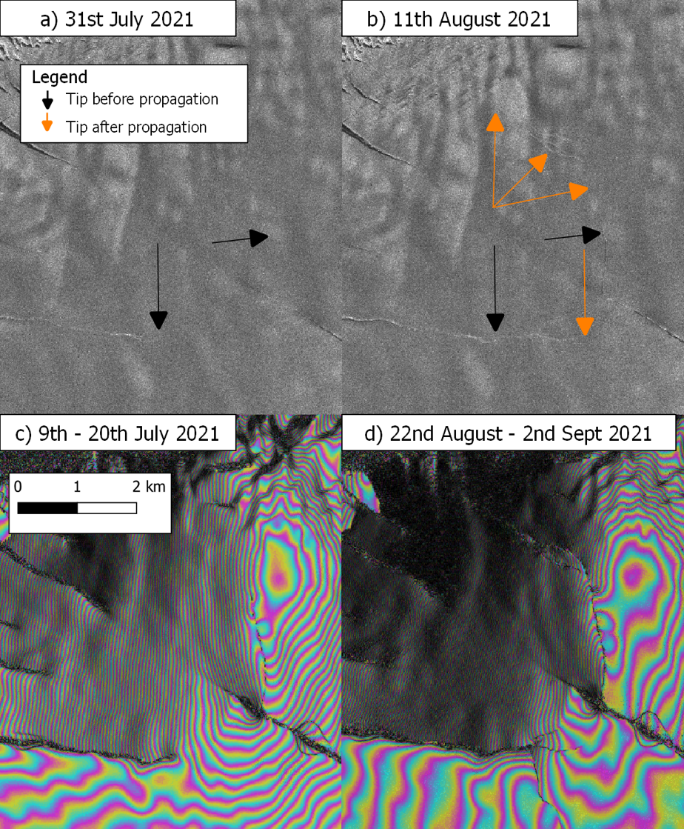


Figure S5: TerraSAR-X backscatter images showing crack growth associated with iceberg collision: a) before and b) after iceberg collision showing extension of surface cracks. c-d) As above with interferograms where fringe discontinuities highlight areas of rift growth.


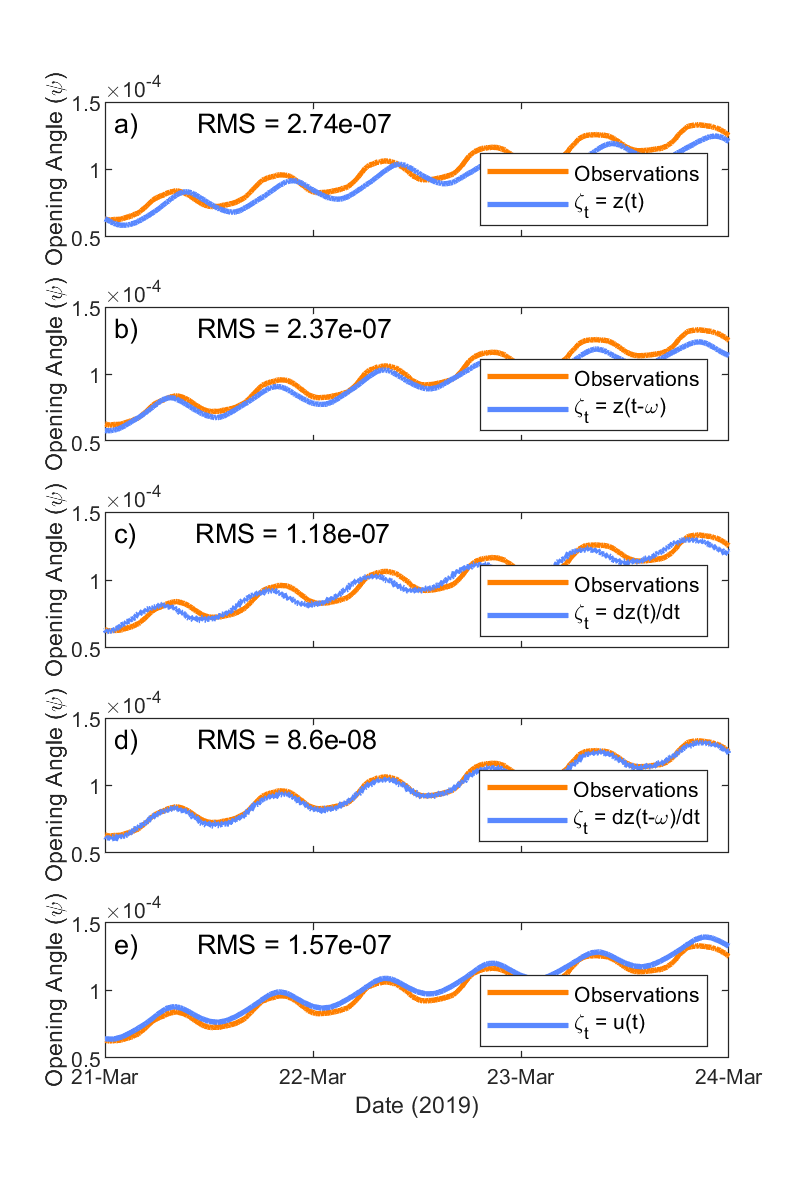


Figure S6: Correlation between opening angle and functions of the tidal amplitude: a) tidal amplitude z, b) tidal amplitude with a time shift of minus 2 hours, c) rate of change of tidal amplitude, d) rate of change of tidal amplitude with a time shift of plus 1.5 hours, e) tidal current in easterly direction from CATS2008 [36].


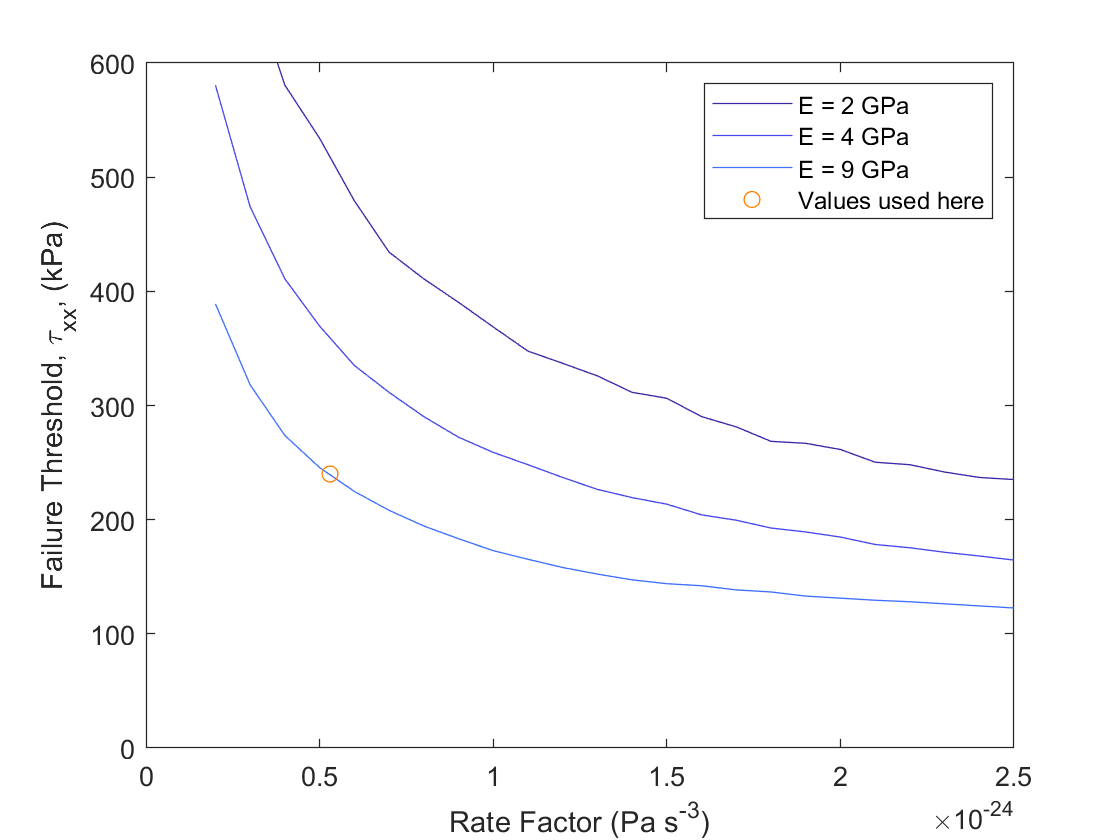


Figure S7: Sensitivity of the estimated failure threshold to changes in rate factor (A) and elastic modulus (E). The orange circle indicates the values of A and E used here to represent conditions on the Brunt Ice Shelf with Poisson Ratio of 0.3. The lines indicate how the derived failure threshold, would be different if different parameters were used.
