# Supplementary material for: Ocean tides trigger ice shelf rift growth and calving
Source: Nat Commun. 2025 Jul 24;16:6697. doi: 10.1038/s41467-025-61796-w (PMC12290053; doi:10.1038/s41467-025-61796-w)
Supplement: Supplementary file 2 — Description of Additional Supplementary Files [file 41467_2025_61796_MOESM2_ESM.docx]

File Name: Supplementary Video 1:

Description: Animation showing the movement of iceberg A-74 including the collision of the iceberg with the Brunt Ice Shelf in August 2021. The iceberg outline at the time of calving is translated and rotated based on GPS data from the two positions indicated.

File Name: Supplementary Video 2:

Description: Animation showing the tidal movement of iceberg A-81 after calving in January 2023. The iceberg outline at the time of calving is translated and rotated based on GPS data from the two positions indicated.
